# Malaria: elimination tale from Yunnan Province of China and new challenges for reintroduction

**DOI:** 10.1186/s40249-021-00866-9

**Published:** 2021-07-21

**Authors:** Heng-Lin Yang, Zulqarnain Baloch, Jian-Wei Xu, Xiao-Dong Sun, Zu-Rui Lin, Yao-Wu Zhou, Xiao-Tao Zhao, Quan Lv, Shi-Yuan Xu, Chun-Li Ding, Qi-Yan Chen, Peng Tian, Kai-Xia Dung, Xue-Shan Xia, Hong-Ning Zhou

**Affiliations:** 1 Yunnan Provincial Key Laboratory of Vector-Borne Diseases Control and Research & Yunnan Innovative Team of Key Techniques for Vector-Borne Disease Control of Yunnan Institute of Parasitic Diseases, Pu’er, 665000 Yunnan China; 2 Faculty of Life Science and Technology, Kunming University of Science and Technology, Kunming, 650500 Yunnan China

**Keywords:** Malaria, Eliminate, Southeast asia, Yunnan, China

## Abstract

**Background:**

Eradication of infectious disease is the sanctified public health and sustainable development goal around the world.

**Main body:**

Three antimalarial barriers were developed to control imported malarial cases, and an effective surveillance strategy known as the “1–3–7 approach” was developed to eliminate malaria from the Chinese population. From 2011 to 2019, 5254 confirmed malaria cases were reported and treated in Yunnan Province, China. Among them, 4566 cases were imported from other countries, and 688 cases were indigenous from 2011 to 2016. Since 2017, no new local malarial case has been reported in China. Thus, malaria has been completely eliminated in Yunnan Province. However, malaria is detected in overseas travellers on a regular basis, such as visitors from neighbouring Myanmar.

**Conclusion:**

Hence, the strategies should be further strengthened to maintain a robust public health infrastructure for disease surveillance and vector control programs in border areas. Such programs should be supported technically and financially by the government to avert the possibility of a malarial resurgence in Yunnan Province.

**Graphic Abstract:**

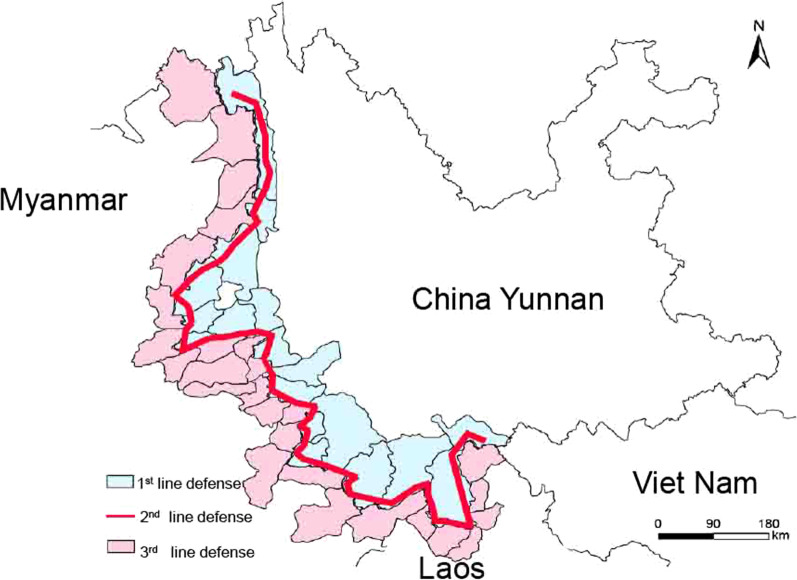

**Supplementary Information:**

The online version contains supplementary material available at 10.1186/s40249-021-00866-9.

## Background

Eradication of infectious disease is the sanctified public health and sustainable development goal around the world. Malaria is an infection caused by the *Plasmodium* parasite and is transmitted by *Anopheles* mosquitoes [1]. According to the World Health Organization (WHO), in 2019, 229 million malarial cases were reported worldwide, with 409 000 deaths. Five African states accounted for 51% of all cases (Nigeria 27%, The Democratic Republic of the Congo 12%, Mozambique 4%, and Niger 3%), and south-eastern Asian states accounted for 3% of global malarial cases [2]. In 1955, China launched a National Malaria Control Program, and policies were implemented to improve irrigation methods, reduce mosquito breeding grounds, use insecticides and use nets for sleeping. Therefore, the total number of malaria cases declined from 30 million in 1955 to 117 000 in 1990. Thus, malaria was successfully controlled in China except in Yunnan Province [3].

Yunnan is a relatively underdeveloped area located in southwestern China and shares 4060 km long porous international borders with malaria-endemic countries such as Lao People`s Democratic Republic (Lao PDR) Myanmar and Vietnam. The tropical monsoon climate produces a generally warm temperature with an average annual rainfall from 600 to 2300 mm. *Anopheles* mosquitoes are widely distributed, and local climatic conditions are favourable for mosquito proliferation and advantageous for *Plasmodium* development. Cross border movement of malaria-infected individuals from endemic areas or countries into Yunnan Province will pose a great risk of malarial resurgence in China [4]. Because of the continuous threat of malarial resurgence, it is important to be highly vigilant against importing malarial cases into the mainland of China.

## Main body

In this article, we explored the prevalence of malaria among immigrants from 2011 to 2019 in Yunnan Province of China and felt proud that there were no indigenous malaria cases. In this study, we evaluated all of Yunnan Province and the border areas of China with Lao PDR and Myanmar. To detect, treat, and control imported malaria cases, three anti-malarial barriers were deployed by dry port quarantine authorities and the Chinese Center for Disease Control and Prevention (China CDC) (Additional file 1: Figure S1): (I) at the 1^st^ barrier, a well-organized joint control system was developed for reports of malaria cases along the international borders between P.R. China, Lao PDR, and Myanmar; (ii) at the 2^nd^ barrier, 68 malarial checkpoints were established to provide screening and consultation with migrants/mobile populations; and (iii) at the 3^rd^ barrier, an efficient health care system was developed in all 25 counties bordering Lao PDR and Myanmar. For laboratory diagnosis, a 5–10 ml venous blood sample was collected from each suspected malarial migrant at the entry point, and a malaria test was performed by microscopic examination of Giemsa-stained blood smears. From 2011 to 2019, a total of 4566 imported cases were reported; 864 cases were foreign nationals, and 3702 cases were returning Chinese nationals. The number of confirmed malaria cases started decreasing, as shown in a sharp decline in cases except in 2015 **(Table ****1****)**, when malaria cases slightly increased to 593. Among 864 foreign nationals with malaria, 859 cases came from Myanmar, and 5 cases came from the rest of the world.

**Table 1:** The total number of Malaria Cases in Yunnan province of China from 2011 to 2019

Year	No. of cases	Classification of parasites						Local	Imported
		Pv	Pf	Po	Pm	Mixed infection^a^	Unclassified^b^		
2011	1533	1011	301	0	5	4	46	397	1136
2012	858	595	175	0	0	5	0	165	693
2013	580	459	106	0	0	3	1	62	518
2014	532	425	95	0	2	4	0	44	488
2015	612	524	86	2	1	2	0	19	593
2016	413	358	53	0	0	2	0	1	412
2017	325	287	34	2	1	1	0	0	325
2018	213	172	30	5	3	0	0	0	213
2019	188	163	21	0	4	0	0	0	188
Total	5254	3994	901	9	16	21	47	688	4566

*Pv* Plasmodium vivax, *Pf* Plasmodium falciparum, *Po* Plasmodium Ovale, *Pm* Plasmodium malariae (Pm)

^a^Mixed infection: multiple malaria species infections

^b^Unclassified: There were cases reported in 2011 and 2013 which cannot be classified. Therefore we mention them as unclassified cases

To detect, treat and control indigenous malaria cases in Yunnan Province of China, we applied an effective surveillance strategy known as the “1–3–7 Approach” [5]. Once a suspected case was reported, a 5–10 ml venous blood sample was taken on day 1, and a malaria diagnosis was performed by using microscopic examination of Giemsa-stained blood smears or rapid malaria dipstick test (RDT) Within 3 days, all positive malaria cases were completely investigated, and classification was performed by the local county Centre of Disease Control (CDC) by confirming the status of imported or locally acquired cases. Within 7 days, the local county CDC completed the investigations and minimized the possible risk of malaria outbreaks.

From 2011 to 2016, 688 indigenous malaria cases were reported in Yunnan Province, China (Table 1). In 2011, 397 indigenous malaria cases were reported in 39 counties of Yunnan Province (Additional file 2: Figure S2); however, due to meticulous control efforts, the number of indigenous cases decreased sharply from 397 cases in 2011 to 1 case in 2016 (Additional file 2: Figure S2). Remarkably, no new indigenous case was reported from 2017 to the present (Table 1).

With globalization and increased international movement, imported malarial cases are becoming a risk of resurgence or reintroduction for countries that are approaching or have achieved eradication [6]. There are different specific challenges for malarial elimination from Yunnan Province, such as regular movement of the border population for their business, jobs, construction, schooling, mining and other activities on both sides of the border [3, 7, 8]. The malaria prevalence is significantly higher among border migrants than among local residents [8]. Therefore, such movements can become a constant threat to bring malaria into Yunnan Province of China.

*Anopheles* species are frequently and widely distributed in Yunnan Province, making the patterns of malaria transmission more complex [9]. Considering the abovementioned facts, malaria management in migrant populations is very difficult. Therefore, strong support from the government and the full cooperation of different sectors are essential to control malaria.

## Conclusions

After persistent efforts, malaria was successfully eliminated from the indigenous population in Yunnan Province. However, imported malaria cases are still identified on a regular basis, particularly in border areas of neighbouring countries, such as Myanmar. Therefore, a continuous massive influx of mobile devices and migrants from Myanmar and other neighbouring countries poses a serious risk for the resurgence of malaria in Yunnan Province. In Yunnan, a relatively strong and effective malaria control system already exists at the prefecture, county, town and village levels. However, insufficient staffing of malaria control programs, the retirement of technical staff, the transfer of malaria control program staff into other programs and a lack of investment from the government have weakened malaria control efforts. Hence, such effective strategies should be further employed to maintain robust public health infrastructures for disease surveillance and vector control programs. The government should provide sufficient technical and financial support to avert the possibility of malaria resurgence in Yunnan Province.

## Supplementary Information

**Additional file 1: Figure S1.** Schematic presentation of the three anti-malaria barriers developed to control imported cases in Yunnan Province.

**Additional file 2: Figure S2.** Clusters of indigenous malaria cases in Yunnan Province, 2011–2016.

## Data Availability

The aggregate data supporting findings contained within this manuscript will be shared upon request submitted to the corresponding author. Identifying patient data will not be shared.

## Abbreviations

Lao PDR: Lao People’s Democratic Republic
China CDC: Chinese Center for Disease Control and Prevention
RDT: Raped malaria dipstick test
